# Challenges of Implementing LLMs in Clinical Practice: Perspectives

**DOI:** 10.3390/jcm14176169

**Published:** 2025-09-01

**Authors:** Yaara Artsi, Vera Sorin, Benjamin S. Glicksberg, Panagiotis Korfiatis, Robert Freeman, Girish N. Nadkarni, Eyal Klang

**Affiliations:** 1 Azrieli Faculty of Medicine, Bar-Ilan University, Zefat 1311502, Israel; yaara.artsi77@gmail.com; 2 Department of Radiology, Mayo Clinic, Rochester, MN 55905, USA; sorin.vera@mayo.edu (V.S.); korfiatis.panagiotis@mayo.edu (P.K.); 3 The Charles Bronfman Institute of Personalized Medicine, Icahn School of Medicine at Mount Sinai, New York, NY 10029, USA; ben.glicksberg@gmail.com (B.S.G.); robert.freeman@mountsinai.org (R.F.); girish.nadkarni@mountsinai.org (G.N.N.); 4 The Windreich Department of Artificial Intelligence and Human Health, Mount Sinai Medical Center, New York, NY 10029, USA; 5 The Hasso Plattner Institute for Digital Health at Mount Sinai, Icahn School of Medicine at Mount Sinai, New York, NY 10029, USA

**Keywords:** large language models, artificial intelligence, clinical practice, clinical decision support

## Abstract

Large language models (LLMs) have the potential to transform healthcare by assisting in documentation, diagnosis, patient communication, and medical education. However, their integration into clinical practice remains a challenge. This perspective explores the barriers to implementation by synthesizing recent evidence across five challenge domains: workflow misalignment and diagnostic safety, bias and equity, regulatory and legal governance, technical vulnerabilities such as hallucinations or data poisoning, and the preservation of patient trust and human connection. While the perspective focuses on barriers, LLM capabilities and mitigation strategies are advancing rapidly, raising the likelihood of near-term clinical impact. Drawing on recent empirical studies, we propose a framework for understanding the key technical, ethical, and practical challenges associated with deploying LLMs in clinical environments and provide directions for future research, governance, and responsible deployment.

## 1. Introduction

Large language models (LLMs), such as OpenAI’s GPT family [1], Google’s Gemini models, and open-source models such as Meta’s LLaMA models [2], are increasingly explored for use in healthcare [3,4]. These systems are designed to process and generate human-like language based on vast training datasets, potentially offering valuable assistance in summarizing clinical notes, explaining test results to patients, or supporting differential diagnoses. However, despite their promise, integration of LLMs into clinical workflows is far from simple.

Language in medicine serves as a means of communication between physicians and as a tool for conveying information to patients. Clinical language carries dense layers of meaning, often combining medical terminology with implied knowledge, culturally shaped metaphors, and patient-specific nuances [5,6]. A single phrase, such as “failure to thrive” or “comfort care,” can encompass an entire trajectory of clinical judgment, ethical consideration, and emotional weight.

LLMs, while technically proficient in generating grammatically sound text, are trained primarily on written corpora and may lack the contextual and relational knowledge that supports real-world medical dialog [7]. Due to the complex, contextual, and culturally embedded nature of medical language, the risks of misinterpretation, bias, and hallucinations are particularly acute. LLMs might misinterpret abbreviations, idioms, or regionally variant medical shorthand, especially when these differ across specialties, institutions, or countries. Furthermore, clinical language is deeply culturally embedded; how illness is described and discussed can vary based on societal norms and local medical culture. Additionally, LLMs may unintentionally reinforce dominant cultural narratives while marginalizing others, thereby perpetuating existing disparities.

For example, HbA1c is reported as % in the USA but mmol/mol in the UK/EU, with different diagnostic thresholds and unit conventions; naïve conversions by an LLM can invert treatment thresholds. Common abbreviations carry different meanings (e.g., OD = “once daily” in the UK but oculus dexter in North America; AF = atrial fibrillation/afebrile). Moreover, code-status terminology diverges across systems (DNR/DNI in the USA vs. DNACPR/ReSPECT in the UK and POLST/MOST in several jurisdictions) [8].

LLMs’ hallucinations, which are confidently incorrect or fabricated outputs, can be dangerous in medicine, where even subtle errors in phrasing may have implications on diagnosis or treatment [9]. Moreover, fabricated references or guideline citations can propagate misinformation through clinical documentation and educational materials, eroding evidence integrity [10]. Because these hallucinations are delivered with the same confident tone as correct content, they are difficult to detect in busy workflows. They can undermine both patient safety and clinician trust in decision-support tools.

Language in clinical settings is performative. The way a diagnosis is delivered, a prognosis is discussed, or consent is obtained is shaped not only by the content of plain text but by the tone, timing, and interpersonal sensitivity [11,12]. LLMs currently struggle with this aspect of communication [13]. At the same time, new models now display rudimentary forms of emotional intelligence, such as detecting patient sentiment and tailoring tone, suggesting that some long-standing communication gaps may narrow quickly [14,15]. As a result, while LLMs can assist in summarizing information or translating jargon into plain language, their limitations demand critical scrutiny when they are tasked with replacing or replicating the communicative roles traditionally held by human clinicians.

### 1.1. From Linguistic Pitfalls to System-Level Risks

Since misread abbreviations, culturally loaded terms, and tone-sensitive phrasing directly shape LLMs’ output, linguistic challenges become system-level patient-safety risks. To avoid the communication pitfalls inherent to LLMs, it is essential to understand and outline the central operational constraints that govern safe deployment in clinical workflows.

Existing literature describes three bodies of work that largely operate in parallel, with little integration. performance benchmarks on synthetic tasks, high-level ethics or regulatory guidance, and narrow case studies or pilots. Each leaves a deployment gap. Benchmarks rarely translate into workflow-safe controls. Ethical frameworks often lack technical pathways and post-deployment monitoring tables and single-site pilots seldom generalize beyond local infrastructure or governance. This perspective addresses that gap by providing an implementation-forward synthesis, contrasting known challenges with the best-supported mitigations.

**Figure 1:**
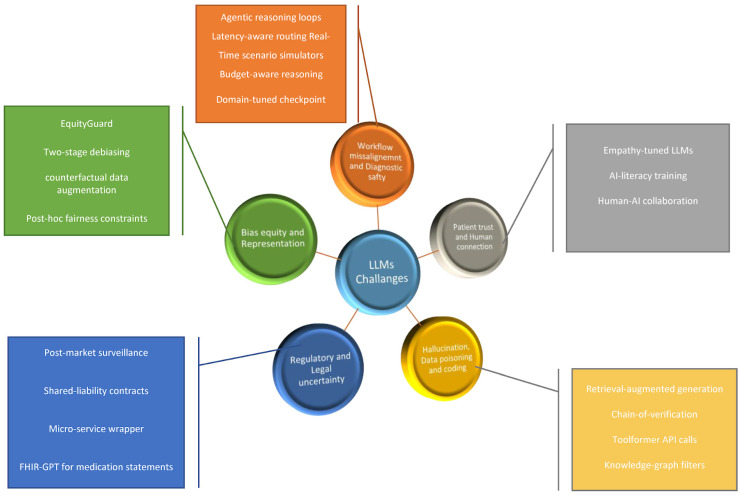
Five implementation challenges for clinical LLMs and targeted mitigations.

Drawing on recently published studies we outline central challenges that LLMs face in implementation in clinical practice, discussing workflow misalignment, bias, and equity, regulatory uncertainty, technical vulnerabilities, and human connection risks. Figure 1 summarizes the five implementation challenge domains we addressed, together with representative mitigation strategies. The deployment barriers domains and their mitigation strategies are summarized in Table 1. along with validation status and model utilization.

### 1.2. Communication Risks Examples

Williams et al. [16] investigated the performance of GPT-4 and GPT-3.5-turbo in generating Emergency Department (ED) discharge summaries and evaluated the prevalence and type of errors across each section of the discharge summary. In a sample of 100 emergency-department encounters, GPT-4-generated discharge summaries contained fewer problems than GPT-3.5; however, errors were still common, with only 33% of GPT-4 summaries being completely error-free vs. 10% for GPT-3.5. Inaccuracies appeared in 10% (GPT-4) vs. 36% (GPT-3.5), hallucinations in 42% vs. 64%, and clinically relevant omissions in 47% vs. 50%. Concrete examples included filling in redacted content as if it were present (e.g., inventing “headache”); adding specialty follow-ups never arranged, inventing ED return precautions or follow-up instructions; leaving out positive exam findings (e.g., a murmur or laceration), ED management details (e.g., specialty consults), or documented symptoms; and misreporting follow-up plans, physical exam findings (e.g., stating a positive exam when negative), imaging as normal when it was not, or social history details.

In a different study, Bischof et al. [17] compared GPT-4 with three established clinical decision support systems (CDSSs), Lexicomp, MediQ, and Micromedex, for recognizing potential drug–drug interactions (pDDIs), using real patient medication lists. The “standard method” identified 280 clinically relevant pDDIs, vs. 80 for GPT-4, indicating substantial under-detection by the LLM compared with CDSSs. GPT-4 markedly under-recognized QTc-prolongation risks (8/80, 10%) compared with the standard method (85/280, 30%). It also produced pharmacology errors (e.g., misinterpreting magnesium supplements as antacids). Prompting specifically for QTc interactions improved detections to 19, but this remained far below the 85 cases flagged by CDSSs. Additionally, they noticed output variability on repeated identical queries, reflecting probabilistic generation and complicating quality control.

## 2. Workflow Misalignment and Diagnostic Safety

While LLMs have demonstrated impressive performance on medical board exams [18,19,20] and synthetic tasks, their behavior in real-world clinical scenarios is often inconsistent. Hager et al. [21] conducted a comprehensive evaluation of leading LLMs, using over 2400 real patient cases from the MIMIC-IV dataset. The models were tested on their ability to interpret evolving clinical data across common abdominal presentations, simulating real-world decision-making.

The results revealed that LLMs underperformed compared to physicians, especially when reasoning through temporally distributed information, such as when clinical information is spread out over time, as seen in patient symptoms evolving, lab results arriving in sequence, or vitals changing over time. While clinicians achieved diagnostic accuracy rates of 88–93%, the best-performing LLM lagged by 16–25 percentage points and failed to request key examinations or laboratory tests. They frequently failed to adhere to established clinical guidelines, misinterpreted lab results without proper contextualization, and showed prompt fragility, producing inconsistent outputs based on minor changes in input phrasing or sequence. These limitations demonstrate that strong performance in synthetic settings does not necessarily translate into clinical reliability.

Similarly, Goh et al. [22] conducted a randomized trial involving 50 physicians from emergency medicine, internal medicine, and family medicine to evaluate the impact of LLMs on diagnostic reasoning. They found no significant improvement in diagnostic accuracy or time spent per case for those using the LLM, despite the LLM performing at 90% accuracy when evaluated independently, showing that clinicians exposed to LLM-generated differential diagnoses did not improve their diagnostic accuracy and were more likely to converge on narrower lists, raising concerns about over-reliance [23]. For now, while LLMs can outperform clinicians on structured diagnostic tasks, their benefit is not automatically realized in practice, which may limit their effectiveness in clinical settings. It should be recognized that the field of generative AI is expanding exponentially, and frontier models continue to improve, utilizing techniques such as reasoning models [24] and agentic frameworks [25].

### Resources, Computation, and Build-vs.-Buy Decisions

Due to new reasoning-tuned and agentic architectures iterating within months rather than years, many of today’s safety gaps may shrink if healthcare-specific fine-tuning and real-time feedback loops are embedded early. However, chain-of-thought and agentic models often increase token counts by two- to four-times, so a response that once arrived in milliseconds can now take 5–10 s, which is unsuitable for high-volume tasks. Latency-aware routing that falls back to a compact classifier for routine requests is emerging as best practice [25].

Lastly, beyond accuracy, institutions must decide whether to license a turnkey cloud model, fine-tune an open-source checkpoint locally, or purchase a task-specific commercial API. Each path entails distinct compute costs, data-security trade-offs, and carbon footprints, making total-cost-of-ownership analyses as important as model benchmarking [24,26].

## 3. Bias, Equity, and Representation

Bias in LLM outputs driven by uneven training data and unbalanced prompt design poses a risk, particularly for marginalized populations [27,28]. Ji et al. [29] investigated how LLMs, such as GPT-4, Gemini, and Claude, respond to socio-demographic cues in two clinical tasks: clinical trial matching and medical question answering.

They found that LLM outputs were significantly influenced by factors such as race, gender, income, housing status, and disability, even when these attributes were irrelevant to the task. The LLMs exhibited substantial disparities in performance across socio-demographic groups, with clinical trial matching accuracy decreasing by up to 10 points for underrepresented populations, and medical question-answering error rates increasing to over 30% for prompts referencing low-income or homeless status. GPT-4 was more consistent but still showed mild bias.

To mitigate this, they developed EquityGuard, a framework that reduces bias by separating task-relevant information from sensitive demographic signals. Applying the EquityGuard mitigation framework significantly reduced these disparities, improving fairness metrics by 28–32% and narrowing performance gaps without sacrificing overall accuracy. Other emerging approaches for bias mitigation are already showing reductions in demographic error gaps in sandbox evaluations. These include counterfactual data augmentation [30,31], two-stage debiasing prompts [32], and post hoc fairness constraints [33]. When considering the implications of LLMs on clinical practice, these results suggest that unmitigated LLMs could exacerbate existing health disparities, particularly in tasks such as trial recruitment or patient communication. Therefore, bias auditing and fairness-aware model development must become standard practice before deploying LLMs in clinical settings.

Omar et al. [34] performed a systematic review of 24 peer-reviewed studies from January 2018 to July 2024 assessing demographic biases in medical LLMs. They found that 91.7% demonstrated measurable bias. Gender bias appeared in 93.7% of the reviewed studies, and racial or ethnic bias in 90.9%. GPT-3.5-turbo predicted lower mortality for White patients (56.5%) than Black patients (up to 62.3%), and GPT-4 recommended advanced imaging less frequently for underrepresented racial groups. While several mitigation techniques, primarily prompt engineering, showed promise, their effectiveness varied, and quantitative results were limited. The near-universal presence of bias underscores LLMs’ risk of perpetuating healthcare disparities unless they are systematically audited and paired with validated fairness interventions.

In a different study by Omar et al. [35], nine LLMs were evaluated using 1000 emergency department vignettes (both simulated and real-world), each repeated with 32 different sociodemographic profiles, resulting in over 1.7 million outputs. Despite identical clinical details, marginalized groups such as Black, unhoused, or LGBTQIA+ patients were more likely to be directed toward urgent care, invasive procedures, or mental health evaluations. For instance, LGBTQIA+ labels triggered mental health assessments six to seven times more often than clinically justified. Conversely, high-income labels increased recommendations for advanced imaging, while low- and middle-income profiles received more basic or no tests at all. These disparities persisted after statistical correction and were consistent across both proprietary and open-source models. Neither model type nor size predicted bias levels.

Importantly, such recommendations had no basis in clinical guidelines, indicating they reflect model-driven bias. In clinical practice, LLMs might inadvertently reinforce inequities by offering different levels of care based on demographic factors rather than medical indications. This stresses the need for bias evaluation and mitigation before deploying LLM-based decision support tools.

## 4. Regulatory and Legal Uncertainty

The current regulatory environment for LLMs in healthcare is lacking [36]. Concerns regarding the deployment of LLMs in current clinical settings include accountability, legal scrutiny, regulation and format interoperability. Ong et al. [37] presented a comprehensive viewpoint on the ethical and regulatory challenges posed by LLMs in medicine. They emphasize key issues, including uncertain data provenance, intellectual property concerns, patient privacy risks due to unclear data-use agreements, and the inherent plasticity of LLMs, which can adapt in unpredictable ways post-deployment.

They argue that under current frameworks in the U.S. and Europe, LLM-based tools offering diagnostic or therapeutic suggestions may fall under the definition of medical devices, yet lack clear regulatory pathways, such as standards for validation, auditability, and post-market surveillance. Legal accountability remains ambiguous. It is unclear whether clinicians, institutions, or model developers bear liability when LLM-driven recommendations result in harm [38]. LLM applications that perform clinical decision-making are likely to be regulated as medical devices; however, few meet the evidence thresholds required for such designation [39]. Moreover, determining liability when an LLM makes an incorrect recommendation remains unresolved, posing legal risks to both clinicians and developers.

They conclude by calling for a structured governance strategy, including transparent documentation of training data, clear consent processes for patients, and continuous model monitoring to ensure responsible integration of LLMs into clinical practice. Regulatory uncertainties hinder the adoption of LLMs, and without clear frameworks for audit, accountability, and safety benchmarking, LLMs risk entering clinical use without adequate protection for patients or providers.

Harshe et al. [40] examined five LLMs, ChatGPT-4o mini, Claude 3.5 Sonnet, Microsoft Copilot, LLaMA 3, and Gemini 1.5 Flash across three ethically charged scenarios: using robotic surgery against patient preference, withdrawing life-sustaining treatment without a surrogate, and acting as a surrogate decision-maker. In the withdrawal scenario, all five LLMs (100%) independently advised seeking a human ethics committee rather than rendering a unilateral decision, underscoring their recognition of authority limits. When asked to serve as a surrogate, four models immediately refused and one deflected, resulting in a refusal rate of 80%. Each model invoked at least two core bioethical principles, most often autonomy and beneficence. However, none cited institutional policy or legal standards, revealing a shared blind spot.

### 4.1. Governance Levers for Safe Use

Although the LLMs listed factors such as patient values and quality-of-life considerations, none could synthesize these into a defensible final judgment without directing users back to human oversight. These findings suggest that current LLMs can help clinicians surface and organize ethical considerations but lack the nuanced contextual reasoning required to replace human surrogates or ethics committees.

Klang et al. [41] discussed the accelerating capabilities of LLMs and other AI systems, arguing that healthcare may be nearing a critical inflection point where machines begin to outperform humans not just in narrow tasks, but in high-level reasoning, synthesis, and complex decision-making. They outline how the line between human and machine judgment may increasingly blur, especially as LLMs demonstrate competencies in interpreting clinical context, supporting longitudinal patient care, and offering recommendations that reflect both medical and ethical considerations [42].

A central theme is a shift from AI as a tool for automation to AI as a potential partner in clinical reasoning. LLMs are beginning to display emergent behavior, synthesizing data across modalities and timelines, identifying patterns invisible to human clinicians, and offering responses that are context-aware and sometimes empathetic in tone. They frame the moment not as a technological inevitability but as an ethical and organizational challenge. This calls for public discourse involving clinicians, ethicists, and regulators to shape how AI systems are designed, evaluated, and integrated into care. Thoughtful governance and interdisciplinary leadership are essential to ensure these technologies remain aligned with human-centered care, clinical integrity, and equitable access.

Addressing this challenge, the FUTURE-AI initiative [43] offers a structured framework for the ethical and responsible deployment of AI in healthcare. Developed by over 100 multidisciplinary experts, the guideline introduces a framework built on six core principles: fairness, universality, traceability, usability, robustness, and explainability. These principles are operationalized into 30 actionable recommendations designed to guide AI development from early design through validation, regulation, deployment, and post-market monitoring.

One of the central ethical concerns addressed is fairness. The guideline emphasizes the need to assess and mitigate algorithmic bias across demographic groups before clinical implementation, calling for pre-deployment bias audits and demographic performance reporting. Universality is another key theme, ensuring that AI tools are culturally and contextually adaptable to different healthcare systems and patient populations, particularly in under-resourced settings.

Traceability and explainability are closely tied to trust and accountability. The guideline emphasizes the importance of transparent documentation of training data, model architecture, and updates over time, enabling external audits and ensuring reproducibility. AI systems should also provide interpretable outputs that clinicians and patients can understand and question.

To complement the FUTURE-AI initiative, the World Health Organization (WHO) published an ethics and governance of AI for Health guidance on LLMs [44]. It translates high-level principles into operational controls for health systems and vendors. It recommends a risk-based approach to oversight and pre-deployment evaluation using representative data with transparent documentation. It also states the importance of human oversight with clearly assigned accountability along with safeguards for privacy, cybersecurity, and intellectual property. Furthermore, it underscores the need for equity auditing to detect and mitigate performance gaps across populations, and continuous real-world monitoring with incident reporting and mechanisms for model update or withdrawal. The guidance also emphasizes truthful communication with patients and disclosure of AI use.

### 4.2. Standards & Interoperability

Tran et al. [36] argued that technical interoperability is a crucial factor in moving generative AI from sandbox to production. The required output standard depends on where the LLM’s data ultimately land. For tasks that stay inside an analytics dashboard or a clinician-facing summarizer, ordinary JSON may be adequate, since a downstream service can translate or simply display the result. In contrast, any workflow that injects data into the transactional heart of the HER, such as placing medication orders, posting lab results or updating allergies, must deliver a payload the interface engine will accept, typically an HL7 v2 message or a FHIR-conformant resource. Thus, the integration rule is conditional, JSON when the data remain in a contained application tier, and conversion to HL7/FHIR whenever the payload must cross the EHR boundary or participate in regulated health-information exchange.

Tran et al. [34] mention in their study several organizations that wrap each LLM call in a micro-service that instructs the model to “return a US Core-conformant FHIR JSON bundle,” validates the response against a profile schema, and logs any failures for clinician review. Deciding on the target standard at design time eliminates costly retro-conversion. Early pilot data show the approach is viable.

Li et al. [45] developed FHIR-GPT, an LLM-powered micro-service that turns free-text medication mentions into fully valid HL7 FHIR medication statement resources. The system reached an overall exact-match rate > 90% against gold-standard annotations. Relative to a multi-component NLP pipeline, FHIR-GPT raised exact-match performance for critical fields by 3% (route), 12% (dose quantity), 35% (reason), 42% (form), and >50% (timing schedule). Almost all generated bundles passed FHIR schema validation on the first attempt, demonstrating that a single LLM, when wrapped in a validation micro-service, can outperform rule-based and deep-learning ensembles

## 5. Hallucinations, Data Poisoning, and Coding Issues

Critical technical vulnerabilities in LLMs that threaten their safe deployment in clinical practice include hallucinations, data poisoning, and coding errors. Hallucinations produce confident but false information [46]. These can lead to boldly stated yet false medical claims, which can cause harm to patients. Data poisoning introduces malicious inputs that corrupt model behavior [47,48], and coding inaccuracies risk undermining administrative workflows and documentation [49]. Together, these issues underscore the need for validation, monitoring, and mitigation strategies before LLMs can be trusted in clinical environments.

Alber et al. [50] conducted a threat assessment to determine the vulnerability of LLMs to data poisoning attacks. They injected 0.001% of medical misinformation into the LLM training dataset, simulating realistic poisoning scenarios. Despite this minimal corruption, harmful completions in response to adversarial prompts increased by 4.8%, even though their performance on standard language benchmarks remained unchanged, highlighting that traditional benchmarks fail to detect poisoned models.

To detect and filter these unsafe responses, the authors developed a mitigation strategy utilizing biomedical knowledge graphs, which achieved high performance with an F1 score of 85.7% (precision 81.3%, recall 91.9%). Notably, the entire poisoning process was low-cost and technically simple, demonstrating that real-world attackers could feasibly compromise LLMs by injecting misinformation into publicly accessible training data sources. Approximately 27.4% of medical concepts in the dataset originate from unmoderated web sources, making them vulnerable to poisoning. Yet standard evaluation frameworks could not flag the resulting errors.

These results have immediate implications for the deployment of clinical AI. They reveal that LLMs can be compromised by small-scale, inexpensive poisoning attacks that elude routine benchmark checks. This calls for stronger source control, continuous output monitoring, and knowledge-graph-based defenses. Tools that appear accurate by conventional metrics may still harbor hidden risks, requiring architects of medical AI systems to implement safeguards to ensure patient safety.

Soroush et al. [51] evaluated GPT-3.5, GPT-4, Gemini Pro, and LLaMA-2-70b on a real-world corpus of over 27,000 unique ICD-9, ICD-10, and CPT codes. GPT-4 showed the highest exact-match accuracy of 46% for ICD-9, 34% for ICD-10, and 50% for CPT (Table 2). Other models underperformed in comparison. Beyond exact codes, LLMs occasionally suggested equivalent or more generalized codes, but these attempts were rare (7–10%) and often still imprecise. They also tracked how code frequency, code length, and description length affected performance. Shorter, more common codes were more likely to be accurately predicted.

In further research, Klang et al. [52] have shown how the use of retrieval augmented generation (RAG) techniques can mitigate hallucinations in medical code generation. Moreover, the study demonstrated that human reviewers favored GPT-4 for accuracy and specificity over provider-assigned codes (*p* < 0.001). This exemplifies the value of exploring various mitigation techniques to enhance LLM outputs in a clinical context. For LLMs to aid in medical administrative workflow, deployment should include domain-specific fine-tuning or retrieval-augmented strategies to ensure accuracy and prevent disruptive errors in healthcare operations.

Interestingly, Dumit et al. [54] argued that hallucinations are not accidental flaws but are inherent to the design of these systems. LLMs generate text by predicting plausible word sequences, rather than verifying facts and producing confident but incorrect outputs. Due to LLMs’ training data, which contains both accurate and erroneous information, they produce plausible-sounding inaccuracies as a natural consequence of their design rather than as avoidable defects.

Their key insight reframes hallucinations as an intrinsic and inevitable characteristic of LLMs. This view challenges the mindset that hallucinations can be eliminated through better data curation or model refinement [55]. Instead, they advocate for a shift in approach by recognizing hallucinations as inherent, rather than accidental. They argue that safety strategies must focus on mitigating their impact, for example, by verifying responses against reliable knowledge bases, rather than removing them entirely.

In recent studies, new strategies to mitigate hallucinations include hybrid retrieval-augmented generation, which anchors responses to external evidence bases [56]. Chain-of-verification prompting, where the model drafts an answer and then explicitly re-checks each factual claim [57], and tool-former architectures that invoke domain-specific application programming interface (APIs) during generation, pulling authoritative facts instead of relying solely on its internal probabilities [58].

Moreover, pairing retrieval grounding with verification chains yields additive gains, suggesting the methods are complementary rather than mutually exclusive [56,57]. Although large-scale clinical trials are still lacking, the rapid progress indicates that iterative model–tool co-design could bring hallucination risk to clinically acceptable levels, especially once effectiveness is benchmarked across multilingual datasets and real-time workflows.

### Detecting and Mitigating Hallucinations

Hallucinations arise from generative next-token prediction [54]. The goal in clinical settings is not elimination but risk reduction through layered safeguards. Farquhar et al. [53] introduced “semantic entropy,” a model-agnostic way to flag likely hallucinations by sampling multiple answers from an LLM, grouping them into semantically equivalent clusters using natural-language inference, and then measuring the entropy of that distribution. When the samples disagree on meaning (high semantic entropy), the initial answer is more likely to be wrong; when they agree (low entropy), it is more trustworthy. Across open-domain QA and long-form generation tasks, semantic entropy consistently outperformed token-level confidence measures for detecting errors and enabled selective answering to boost overall accuracy. This approach requires no retraining and works across different models, readily usable as a safety gate in clinical workflows.

To mitigate hallucinations, another strategy is RAG, which grounds answers in a pre-approved set of sources, reducing unsupported claims and adding provenance. Unlike fine-tuning, RAG does not alter model weights but composes answers from the retrieved sources, enabling safer refusal when evidence is absent [59]. Another mitigation technique is verification prompting, such as chain-of-verification. The model fact-checks itself before it finalizes an answer, instead of accepting a single long response that may contain hallucinations. Dhuliawala et al. [57] showed that Chain-of-Verification (CoVe) substantially cuts hallucinations. On Wikidata list queries, precision more than doubled with CoVe (0.17→0.36) while false entities fell (2.95→0.68).

Additional mitigation techniques include confidence calibration, which aims to align a model’s stated confidence with its actual likelihood of being correct, using uncertainty signals (e.g., token-level variance, entropy, or disagreement across multiple sampled answers) to temper overconfident outputs, selective abstention then operationalizes these signals by allowing the system to withhold an answer or automatically route to retrieval or human review whenever confidence falls below a predefined threshold. Together, they reduce false certainty and lower the risk of high-consequence errors [60].

Tool-assisted reasoning [58] limits guessing for factual subproblems. The LLM externalizes verification (via APIs) rather than relying on the model’s internal weights alone. Finally, human-in-the-loop review remains essential. Routing low-confidence outputs to clinicians and monitoring post-deployment via prospective audits. Collectively, these measures shift LLM use from open-ended free text to verifiable outputs with documented provenance and explicit escalation paths.

## 6. Patient Trust, Human Connection, and Over-Reliance

Even when LLMs function effectively, patient trust often remains a barrier. Past studies have shown that patients can express concern about AI systems lacking empathy, emotional nuance, and cultural understanding [61,62] or that LLMs may not be seen as credible or compassionate participants [13]. However, Sorin et al. systematically reviewed the literature and demonstrated that LLMs already exhibit “elements of cognitive empathy, recognizing emotions and providing emotionally supportive responses in various contexts” [63]. As LLMs continue to advance, it is expected that their cognitive empathetic capabilities will also keep improving [64].

Choudhury et al. [65] investigated the evolving relationship between clinicians and LLMs in healthcare. The increasing reliance on AI-generated content may trigger a feedback loop that could impede model accuracy and human expertise. As LLMs are trained increasingly on their outputs rather than diverse human-generated data, they risk entrenching biases and diminishing performance over time. This self-referential loop may also contribute to the deskilling of clinicians, who might underutilize critical thinking in favor of convenience [21,66].

While expert users can mitigate these risks by critically verifying AI output, blind trust followed by prior positive experiences can lead to automation bias and compromised decision-making. This emphasizes the importance of transparency, clinician oversight, and legislative safeguards, preserving clinician competence while leveraging AI to improve efficiency and care quality.

A potential risk of replacing clinicians with automated responses is the erosion of the doctor-patient relationship. Even a simple gesture, such as a tap on the shoulder, has a calming, grounding effect, conveying empathy and reassurance in a way that words alone often cannot [67]. The subtle nuances of human interaction play a role in building trust and emotional safety in clinical encounters [68]. Replacing these with automated responses risks flattening the therapeutic relationship. As LLMs become more embedded in clinical practice, preserving opportunities for human connection should remain a priority.

### Real-World Deployments and Early Outcomes

The real-world use of LLMs in clinical settings is emerging but remains in its early stages [69]. Major EHR vendors have begun embedding generative AI features in production [70], with early site reports indicating reduced documentation time. However, these deployments remain heterogeneous and largely vendor-reported. Large-scale, independent evaluations of clinical text generation quality, safety, and downstream outcomes are still limited. Accordingly, current activity is confined to supervised pilots within defined workflows. Health systems are piloting LLMs for assisting in tasks such as drafting progress notes and discharge instructions, triaging inbox messages, and converting free-text into interoperable formats for downstream systems [45]. Specialty domains are also exploring the pragmatic adoption of these technologies.

Cersosimo et al. [71] conducted an exploratory dialog with ChatGPT, with a particular focus on arrhythmia management and cardiac electrophysiology. They assessed the LLM’s capability in interpretation, arrhythmia detection, and procedural guidance. Early reports describe LLMs supporting patient communication, summarizing rhythm-monitoring data, and generating draft procedural documentation.

These use cases are better suited to supervision-heavy workflows rather than autonomous decision-making. This is a repeated theme, where value appears first in bounded tasks with clear ground truth and automated validation. Prospective studies of clinical outcomes, safety signals, and equity impacts are still sparse. Taken together, these early deployments justify continued experimentation, with rigorous governance, standards-based interfaces, and prospective evaluation.

To complement the findings in our perspective, Table 2 compares and summarizes results for commonly used LLMs across clinical tasks. Across studies using real-world or realistic inputs, frontier general-purpose models and strong open models show mixed performance. Strong language competence does not uniformly translate into diagnostic accuracy or adherence to guidelines, whereas tightly scoped, schema-constrained tasks show higher reliability. These contrasts reinforce the central claim that performance is task- and integration-dependent, not model-brand dependent.

## 7. Future Directions

### Accountability and Escalation Paths

LLM deployments are inherently dynamic. New checkpoints, updated toxicity filters, and revised alignment layers appear every few weeks, altering behavior and safety profiles. One-time validation is therefore inadequate. Hospitals are adopting modern machine-learning operations (MLOps) for LLM workflows that run every fresh checkpoint through an automated battery of bias, dosage-safety, and FHIR-schema tests, conduct shadow deployments that log, but do not display, the candidate model’s outputs alongside the incumbent system, and roll out canary releases with real-time regression monitors and automatic rollback triggers. Such pipelines avert the cycle of outdated model before deployment issue, as documented by Klang et al. [38]

Given the pace of model evolution, assessment mechanisms must be updated continuously to reflect new architectures and mitigation tools. For safe and effective clinical deployment, evaluation frameworks should incorporate real-world clinical use cases, emphasizing fairness, safety, and transparency, as well as bias auditing, scenario-specific benchmarking, and post-integration monitoring. Future research should focus on prospective research and patient-facing studies, measuring trust, satisfaction, and outcomes.

Clear accountability mechanisms are equally important. LLM-assisted decisions should be traceable to a named clinician or team, as well as institution-level AI safety policies. Models should be published with version histories and undergo independent performance audits. Interdisciplinary governance should guide system design.

Future LLM integration should prioritize human-AI collaboration. In order to ensure responsible adoption across diverse healthcare settings, investing in explainable models, training data that is culturally sensitive, and system design that preserves human oversight is imperative.

## 8. Limitations

This perspective is based on a purposive sample of recent, peer-reviewed literature. Our study selection inevitably reflects the authors’ judgment. The selected studies are limited to English-language sources; important evidence published in other languages may therefore be underrepresented. In addition, the field of clinical LLM research is evolving exponentially; new model capabilities, regulatory guidance, and real-world data keep emerging, which could refine or contradict the challenges outlined in this perspective.

Finally, most of the cited studies rely on simulated vignettes or retrospective datasets, so their findings may not fully capture performance in live clinical environments. These limitations underscore the need for continuous evidence updates and prospective evaluations as LLM technology and policy advance fast.

## 9. Conclusions

LLMs represent a significant advance, with the potential to transform clinical decision-making, documentation, education, and patient communication in the near future. However, current limitations in accuracy, equity, interpretability, and regulation mean they cannot yet be safely or equitably integrated into clinical workflows without careful oversight. Probably more than any previous medical technology, LLMs must be evaluated not only for their technical performance but for their fit within the complex moral, social, and regulatory ecosystems of healthcare.

## Figures and Tables

**Table 1:** Current Risk Domains, Limitations and Fast-Evolving Mitigations for Clinical LLMs.

Risk Domain	Clinical Impact	Leading Mitigation Technique	Validation Status	Models Tested in Studies	Limitations
Workflow misalignment & diagnostic safety	Missed/delayed diagnoses Unnecessary labs/imaging Non-adherence to guidelines latency when large reasoning chains are invoked Budget overruns Delayed deployment	Agentic reasoning loops Latency-aware routing Real-time scenario simulators Token-budget-aware reasoning Local inference on smaller domain-tuned checkpoint	Single-site simulation pilots Sandbox cost-profiling Early MLOps pilots	GPT-4 Gemini 1.5 Sequential-Diagnosis Llama-2 agents Token-Budget-Aware Llama-2	Retrospective/simulated cases Limited patient-outcome data Prompt fragility & variable temporal reasoning across setups Heterogeneous clinician–LLM interfaces
Bias and Equity	Unequal triage imaging, or trial-matching recommendations for under-represented groups	EquityGuard Two-stage debiasing counterfactual data augmentation Post hoc fairness constraints	Sandbox + clinician pilots	GPT-4 Claude 3.5 Gemini	Demographic labels may not reflect chart-level complexity Sparse intersectional analyses
Governance, regulatory & legal	Unclear liability Data-provenance gaps Privacy risk Unsafe orders (JSON mis-mapped to HL7/FHIR)	Post-market surveillance Shared-liability contracts Micro-service wrapper FHIR-GPT for medication statements	Early “live” audits & draft policy frameworks Sandbox	Applied across LLMs in general FHIR-GPT (GPT-4-core) Gemini 1.5	Recommendations are nonbinding Limited post-market surveillance in practice Accountability remains unsettled
Technical vulnerabilities (hallucination, data poisoning, coding)	Confident false outputs Poisoned outputs Billing & quality-reporting errors	Retrieval-augmented generation Chain-of-verification Toolformer API calls Knowledge-graph filters	Controlled benchmarks & limited production pilots	GPT-4 LLaMA-2-70B	Benchmark improvements may not translate to live systems RAG/tooling introduces integration and maintenance burden
Trust and Human Connection	Reduced perceived empathy Automation bias Clinician deskilling	Empathy-tuned LLMs AI-literacy training Human-AI collaboration	Vignette studies & patient-survey pilots	GPT-4o mini Claude Sonnet	Empathy measures are text-based proxies long-term effects not yet quantified

Validation status: sandbox—offline or retrospective/synthetic evaluation without patient-facing use; pilot—limited prospective use or shadow mode in clinical settings; live audit—production deployment with post-deployment monitoring and incident reporting; Model-agnostic—controls applicable across model families. Abbreviations: RAG: retrieval-augmented generation; FHIR: Fast Healthcare Interoperability Resources; MLOps: Machine learning operations.

**Table 2:** Comparative Evidence of Capabilities and Limitations Across Models in Healthcare Tasks.

Study	Models Compared	Task & Inputs	Metric(s)	Key Finding
Hager et al. [21]	LLMs based on Llama 2 vs. clinicians	4 common abdominal presentations from MIMIC-IV	Diagnostic accuracy Test ordering Guideline adherence	Physicians 88–93% accuracy Best LLM lagged by ~16–25% Missed key tests Prompt fragility & guideline deviations noted
Goh et al. [22]	GPT 4-assisted vs. control physicians	Solving clinical vignettes; LLM also evaluated alone	Diagnostic accuracy Time per case	LLM suggestions did not improve accuracy or time Narrowed differentials
Li et al. [45]	Instruction-tuned LLM (FHIR-GPT) vs. ensemble NLP pipelines	Convert clinical text to HL7 FHIR Medication Statement	Exact-match against gold FHIR	>90% exact-match +3–50% improvement of exact match rates of existing NLP pipelines
Soroush et al. [51]	GPT-3.5 GPT-4 Gemini Pro LLaMA-2-70B	Medical code querying over >27 k ICD-9/ICD-10/CPT descriptions	Exact-match code accuracy	GPT-4: 34–50% exact-match; frequent near-misses and fabrications → not deployment-ready
Klang et al. [52]	GPT-4 + retrieval vs. provider codes	ED ICD-10-CM coding with RAG evidence; human review	Reviewer preference Error patterns	GPT-4 + RAG codes preferred over provider-assigned codes (*p* < 0.001) RAG reduced hallucinated codes and improved specificity
Bischof et al. [17]	GPT vs. commercial CDSS	Drug–drug interaction assessment from medication pairs	Sensitivity/specificity Error analysis	CDSS outperformed for severe DDIs GPT missed critical interactions Hallucinated contraindications Unsafe without oversight
Ji et al. [29]	GPT-4 Gemini Claude (sub-versions not specified)	Trial matching & medical Q&A with sociodemographic cues	Accuracy/fairness Group gaps Mitigation impact	Demographic attributes shifted outputs Trial-match accuracy drops up to 10% Q&A errors >30% for low-income/homeless cues EquityGuard cut disparity metrics by 28–32% without harming overall accuracy
Omar et al. [35]	GPT-4o Llama-3.1-70B Llama-3.1-8B Gemma-2-27B-it Gemma-2-9B-it Qwen-2-72B Qwen-2-7B Phi-3-medium-128k-instruct Phi-3.5-mini-instruct	1000 ED vignettes × 32 profiles (1.7 M outputs)	Recommendation rates vs. guidelines Disparity analysis	Marginalized labels drove excess mental-health or invasive workups High-income labels → more advanced imaging Effects persisted across models
Harshe et al. [40]	GPT-4o mini Claude 3.5 Sonnet Copilot LLaMA-3 Gemini 1.5 Flash	Ethics scenarios	Refusal/deflection rate Principle invocation	100% advised human ethics review in withdrawal case 80% refused acting as surrogate Models cited autonomy/beneficence but not local policy/law Appropriate deferral to humans
Farquhar et al. [53]	Model-agnostic detection method	Hallucination detection via semantic entropy on QA/knowledge tasks	AUROC/precision-recall for error detection Abstention utility	Semantic entropy outperformed log-prob and self-reported confidence for detecting wrong answers Enables selective abstention to reduce false claims

Abbreviations: RAG: retrieval-augmented generation; ICD-9/ICD-10-CM: International Classification of Diseases, Ninth/Tenth Revision, Clinical Modification; CPT: Current Procedural Terminology; FHIR: Fast Healthcare Interoperability Resources; HL7: Health Level Seven; CDSS: clinical decision support system; DDI: drug–drug interaction; AUROC: area under the receiver operating characteristic curve.

## Data Availability

All data generated or analyzed during this study are included in this published article.
